# Bar Adsorptive Microextraction Coated with Carbon-Based Phase Mixtures for Performance-Enhancement to Monitor Selected Benzotriazoles, Benzothiazoles, and Benzenesulfonamides in Environmental Water Matrices

**DOI:** 10.3390/molecules25092133

**Published:** 2020-05-02

**Authors:** Samir M. Ahmad, Bruno B.C. Calado, Mariana N. Oliveira, Nuno R. Neng, J.M.F. Nogueira

**Affiliations:** 1 Centro de Química Estrutural, Faculdade de Ciências, Universidade de Lisboa, 1749-016 Lisboa, Portugal; samir.marcos.ahmad@gmail.com (S.M.A.); brunobotocalado@gmail.com (B.B.C.C.); mariananetoliveira@hotmail.com (M.N.O.); 2 Departamento de Química e Bioquímica, Faculdade de Ciências, Universidade de Lisboa, 1749-016 Lisboa, Portugal

**Keywords:** BAµE, carbon-based sorbents, phase mixtures, benzotriazoles, benzothiazoles, benzenesulfonamides, HPLC-DAD, Environmental water matrices

## Abstract

In the present work we propose, for the first time, bar adsorptive microextraction coated with carbon-based phase mixtures, followed by microliquid desorption and high performance liquid chromatography-diode array detection (BAμE-μLD/HPLC-DAD) analysis, to enhance the performance of the determination of traces of benzotriazoles (BTRs), benzothiazoles (BTs), and benzenesulfonamide derivatives (BSDs) in environmental water matrices. Assessing six carbon-based sorbents (CA1, CN1, B test EUR, SX PLUS, SX 1, and R) with different selectivity properties allowed us to tailor the best phase mixture (R, 12.5%/CN1, 87.5%) that has convenient porosity, texture, and surface chemistry (pH_PZC,mix_ ~6.5) for trace analysis of benzenesulfonamide, 1-hydroxybenzotriazole, 1*H*-benzotriazole, 5-methyl-1*H*-benzotriazole, benzothiazole, and 1,3-benzothiazol-2-ol chemicals in aqueous media. Optimized experimental conditions provided average recoveries ranging from 37.9% to 59.2%, appropriate linear dynamic ranges (5.0 to 120.0 µg L^−1^; *r*^2^ ≥ 0.9964), limits of detection between 1.0 and 1.4 μg L^−1^, and good precisions (relative standard deviation (RSD) ≤ 9.3%). The proposed methodology (BAμE(R, 12.5%/CN1, 87.5%)-μLD/HPLC-DAD) also proved to be a suitable sorption-based static microextraction alternative to monitor traces of BTRs, BTs, and BSDs in rain, waste, tap, and estuarine water samples. From the data obtained, the proposed approach showed that the BAμE technique with the addition of lab-made devices allows users to adapt the technique to use sorbents or mixtures of sorbents with the best selectivity characteristics whenever distinct classes of target analytes occur simultaneously in the same application.

## 1. Introduction

Benzotriazoles (BTRs), benzothiazoles (BTs), and benzenesulfonamide derivatives (BSDs) are high production chemicals with varied applications both in industry and at home [1]. BTRs are generally used as anti-corrosion additives in dishwashing detergents, antifreeze products, and brake fluids. Other applications include ultraviolet stabilizers, dyes, and fungicides [2]. BTs are also used as anti-corrosion additives as well as vulcanization accelerators in rubber production, biocides in paper and skin making, as well as in the treatment of other industrial products such as herbicides and fungicides [3]. Finally, BSDs are extensively applied as plasticizers, intermediates in the synthesis of sweeteners, and may also be metabolites of corrosion-inhibiting agents and disinfectants [1]. The widespread use of these compounds, associated with their high-water solubility and some resistance to conventional wastewater treatment, has resulted in their detection in a variety of aqueous matrices, including surface water, atmospheric water, drinking water, as well as primary and secondary wastewater. Additionally, these products are considered as emerging contaminants and have recently been classified as toxic to aquatic organisms, which may cause long-term adverse effects [4]. For these reasons, there is need for the implementation of modern analytical approaches to monitor traces of these compounds in environmental matrices.

Several analytical methods have been proposed for the determination of these compounds in environmental water matrices [1,5,6,7], including microextraction-based techniques such as dispersive liquid-liquid microextraction (DLLME) [8], air assisted liquid-liquid microextraction (AALLME) [9], stir bar sorptive extraction (SBSE) [10], and solid phase microextraction (SPME) [11]. Nevertheless, these technologies present recovery and reproducibility limitations, since the most commonly used sorbents/solvents are devoted to the enrichment of semi-polar to non-polar compounds, of which BTRs, BTs, and BSDs are not (−1.74 ≤ log D ≤ 2.49; 2.0 ≤ matrix pH ≤ 11.0 [12]; Table S1). The log D values represent the correction in log *K*_o/w_ values for the p*K*_a_ of each compound by quantifying the amount of both the ionized and non-ionized forms in octanol and water.

In the last decade, bar adsorptive microextraction (BAμE) has been reported as an alternative enrichment technique that uses activated carbons (ACs) as coating sorbent phases and has demonstrated great performance for trace analysis of polar compounds from aqueous media [13,14,15,16,17,18]. One of the great advantages of BAμE techniques over other microextraction-based methodologies is that the devices are easily lab-made; they can be prepared in any analytical laboratory. Nevertheless, there are many types of commercial ACs that present very different physicochemical properties (e.g., porosity, texture, and pH of the point of zero charge, pH_PZC_) that can affect both the selectivity and the sensitivity, particularly when distinct classes of target analytes occur together in the same sample.

In this work, we propose, for the first time, the use carbon-based phase mixtures in order to enhance the selectivity and the performance of the BAμE technique for trace analysis of BTRs, BTs, and BSDs (benzenesulfonamide, BSA; 1-hydroxybenzotriazole, OHBT; 1*H*-benzotriazole, BT; 5-methyl-1*H*-benzotriazole, MeBT; benzothiazole, BTh; and 1,3-benzothiazol-2-ol, OHBTh) in environmental water matrices. The enrichment stage was followed by microliquid desorption and high performance liquid chromatography-diode array detection (BAμE-μLD/HPLC-DAD) analysis. The proposed methodology was fully optimized and validated in terms of precision, linear dynamic range, and detection limits. The comparison with other microextraction-based methodologies reported in the literature is also addressed.

## 2. Results and Discussion

### 2.1. Instrumental Operating Conditions

The HPLC-DAD conditions were evaluated by considering the UV/vis spectral data for the detection of each analyte (BSA, OHBT, BT, MeBT, BTh, and OHBTh) as well as the characteristics of the retention time and resolution. Since a wavelength of 210 nm maximized the S/N for all the compounds under study, it was selected for further assays. The best chromatographic conditions (Section 2.4) were obtained by manipulating the eluent gradient, column temperature, and injection volume, resulting in a suitable chromatographic performance (Table S2). The linearity was calculated by injecting six standard solutions having concentrations from 250.0 to 5 000.0 µg L^−1^, where it was possible to obtain determination coefficients (*r*^2^) higher than 0.998 for the target analytes. Limits of detection (LODs) and limits of quantification (LOQs) ranging from 10.0 to 100.0 µg L^−1^ and from 33.0 to 330.0 µg L^−1^ were achieved for the target analytes, respectively. Assays performed to assess the instrumental precision resulted in a relative standard deviation (RSD) lower than 4.8%.

### 2.2. Optimization of the BAµE-µLD Efficiency

We started by establishing the experimental conditions that would maximize the microextraction efficiency for the six target analytes (BSA, OHBT, BT, MeBT, BTh, and OHBTh) through BAµE-µLD by optimising the selectivity of the coating phase, equilibrium time, agitation speed, pH, polarity, and ionic strength for the microextraction stage, as well as the solvent type and desorption time for the back-extraction stage, in accordance with previous works [19,20].

#### 2.2.1. Selection of the Carbon-Based Phase

For the sorbent coatings selection, several assays were performed through BAµE using different carbon-based phases to achieve the best selectivity to recover the target analytes from aqueous media. Therefore, six commercially available AC materials (CA1, CN1, B test EUR, SX PLUS, SX 1, and R) were chosen for coating the BAμE devices since in addition to having particular porosity, texture, and surface chemistry, they can promote effective electrostatic/dispersive, π-π, and hydrophobic interactions with organic compounds, as was already demonstrated in previous reports [15,17,21,22,23,24,25].

**Figure 1:**
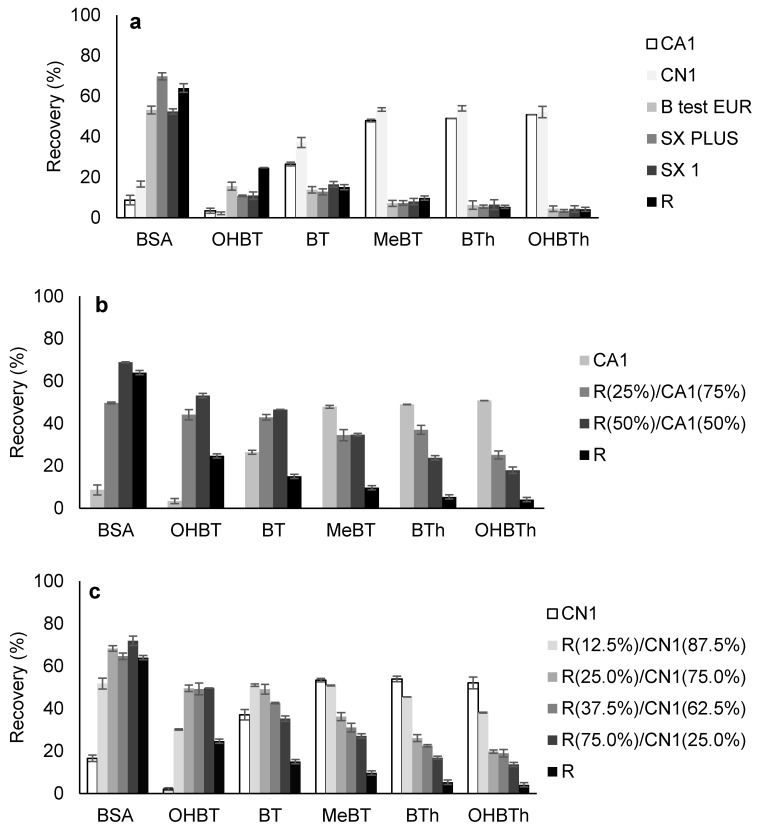
Effect of sorbent selectivity using neat activated carbons (ACs )(**a**), R/CA1 (**b**), and R/CN1 (**c**) mixtures as coating phases for the microextraction recovery of BSA, OHBT, BT, MeBT, BTh, and OHBTh from aqueous media obtained by BAµE-µLD/HPLC-DAD. The error bars represent the standard deviation of three replicates.

Starting with standard conditions (microextraction stage: 16 h (1000 rpm), pH 5.5; back-extraction stage: MeOH (100 µL), 30 min under sonication treatment), a selective differentiation can be clearly observed in Figure 1a. From the data obtained, we can state that CA1 and CN1 phases are very selective for BT, MeBT, BTh, and OHBTh analytes, whereas the remaining sorbents are better at retaining BSA and OHBT, although to a much lesser extent for the latter. As a general trend, the CA1 and CN1 phases suggest greater selectivity for the less polar analytes. For the R, B test EUR, SX Plus, and SX1 phases, the results are the reverse, i.e., the average recovery yields increase for the more polar compounds (BSA and OHBT).

The first set of results were expected since CN1 and CA1 are carbon-based sorbents, which present acidic to neutral characteristics (2.2 < pH_PZC_ < 6.4; Table S1) and have been shown to have good selectivity in other applications, particularly in recovering medium to low polarity compounds from aqueous media [16,17,26]. At pH 5.5, BSA and OHBT present strong polar characteristics (0.58 < log D < 0.61; Table S1), resulting in lower electrostatic/dispersive interactions with the sorbent phases due to the charge formed at the materials’ surface. On the other hand, for the other coating phases that present neutral to basic characteristics (6.0 < pH_PZC_ < 8.4), the interactions would be favoured. For BT, MeBT, BTh, and OHBTh analytes that present smooth polar characteristics (1.81 < log D < 2.49; Table S1), an opposite situation is observed, although to a much lesser extent for BT.

In short, from the data achieved in Figure 1a and since CN1, CA1, and R phases presented the best compromise for all six analytes under study, we can postulate that the combination of these carbon-based sorbents would improve the overall selectivity, as was previously reported by using BAμE devices coated with polymer-based phases [19].

Figure 1b,c shows the average recovery yields obtained by using BAμE devices coated with different phase mixtures of R plus CA1 and R plus CN1, respectively. Firstly, it must be noted that the results obtained for each individual AC phase is consistent with those obtained before (Figure 1a), showing the remarkable reproducibility of this analytical technology. Secondly, the proportion of R/CA1 and R/CN1 phase mixtures increases or decreases the overall average recovery yields following the same pattern. In Figure 1b, as the rate of R/CA1 phase mixture increases, the selectivity for the more polar compounds (BSA and OHBT) also increases, but the recovery of the less polar compounds (BT, MeBT, BTh, and OHBTh) decreases. A similar pattern is also observed in Figure 1c, for the assays performed with R/CN1 phase mixture.

The data obtained clearly showed that by using different combinations and rates of distinct coating phases for BAμE, the selectivity can be tailored to a particular compound or group of compounds, depending essentially on the nature of the carbon-based materials and the target analytes involved. Therefore, if a careful survey is made in the graphic of Figure 1c, the R(12.5%)/CN1(87.5%) phase mixture, having appropriate porosity, texture, and surface chemistry with predominant neutral characteristics (pH_PZC,mix_ ~6.5), presents the best compromise for the selective microextraction of all the six target analytes from aqueous media.

#### 2.2.2. Back-Extraction Stage Conditions

Next, we continued with the back-extraction optimization process, which included the selection of the desorption solvent and sonication time. Therefore, solvents such as MeOH, can, and a mix (MeOH/ACN, 1/1; *v*/*v*) as well as several desorption times (30, 45, and 60 min) were assayed to evaluate the back-extraction performance. The results demonstrate (Figure S1) that the solvent mix using 45 min of sonication showed the best liquid desorption performance, with negligible advantages using longer periods of time.

#### 2.2.3. Microextraction Stage Conditions

**Figure 2:**
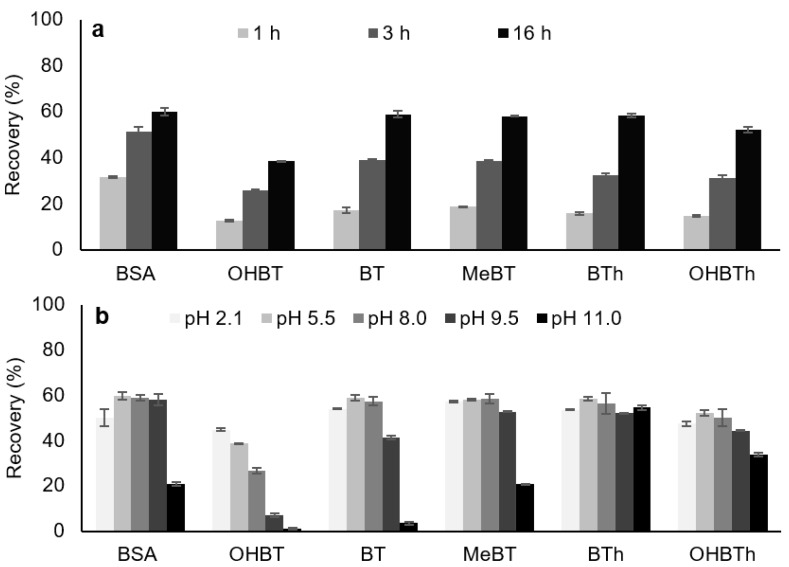
Effect of microextraction time (**a**) and matrix pH (**b**) on the microextraction recovery of BSA, OHBT, BT, MeBT, BTh, and OHBTh from aqueous media obtained by BAµE(R, 12.5%/CN1, 87.5%)-µLD/HPLC-DAD. The error bars represent the standard deviation of three replicates.

Several parameters can affect the microextraction efficiency, as this process is based on equilibrium of the analytes between the bulk aqueous solution and the sorbent phase. Therefore, the kinetics process is affected by important parameters such as the equilibrium time and stirring speed. Three agitation speeds (750, 1000, and 1250 rpm) and equilibrium times between 1 and 16 h were assayed. The results obtained (Figure S2) showed that no significant differences in the overall recovery yields were observed. Furthermore, Figure 2a depicts that 16 h are needed to maximize the microextraction for the target compounds. Although this is a substantial microextraction period, we decided to fix this parameter for further experiments, since the BAµE technique can be performed overnight without any special requirements.

During our studies, sample matrix characteristics were also investigated in order to maximize the methodology efficiency for the analytes under study. For this reason, the effect of matrix pH, ionic strength, and polarity were also assayed. The results obtained demonstrate that the matrix pH greatly influences the recovery of the studied compounds (Figure 2b) as most become ionized at higher pH values (Table S1), which can hinder the microextraction process but limiting π-π or hydrophobic interactions. Once a matrix pH of 5.5 resulted in higher extraction efficiency, this value was chosen for further studies. According to the literature, the “salting-out” effect consists of decreasing the solubility of the analytes in order to force them towards the sorbent phase. On the other hand, the efficiency yields are conditioned many times by the “wall-effect”, in which the analytes can be adsorbed on the sampling glass flask and this can be overcome by modifying the polarity matrix. Thereby, the ionic strength and polarity were also modified through the addition of NaCl (0%, 5%, 10%, 15%, and 20%; *w*/*v*) and MeOH (0%, 5%, 10%, and 15%; *v*/*v*) onto the matrix media, respectively. The results demonstrate that the progressive addition of MeOH or salt (Figure S3) significantly decreased the recovery yields of all analytes and, therefore, both were discarded.

From the data obtained, the fully optimized experimental conditions were (1) microextraction stage: mixture phase (R, 12.5%/CN1, 87.5%), 16 h (1000 rpm), pH 5.5 and (2) back-extraction stage: mix (100 µL), 45 min under sonication.

### 2.3. Validation Assessment

After the optimization process, we proceeded with the validation assays for the BAµE(R, 12.5%/CN1, 87.5%)-µLD/HPLC-DAD methodology. Table 1 presents the data obtained in the present work. The sensitivity was checked through the LODs (1.0–1.4 µg L^−1^) and LOQs (3.3−4.6 µg L^−1^), calculated with an S/N of 3/1 and 10/1, respectively. The present methodology was also evaluated through intraday and interday repeatability assays and calculated as RSD of the recovery yields of five and nine assays, respectively. Interday repeatability assays were carried out as three replicates a day in three consecutive days. Intraday repeatability assays consisted of five replicates performed in the same day, with a spike at the 20.0 µg L^−1^, where good results were obtained (RSD ≤ 9.3%). Assays were also performed in ultra-pure water having concentrations ranging from 5.0 to 120.0 µg L^−1^, where convenient linearity and determination coefficients (*r*^2^ ≥ 0.9964) were obtained for all analytes under study.

### 2.4. Comparison with Other Microextraction-Based Methodologies

In the present work, we decided to compare the proposed methodology (BAµE(R, 12.5%/CN1, 87.5%)-µLD/HPLC-DAD) with other microextraction-based approaches proposed in the literature (Table 2), including SPME [11,27], SBSE [10], DLLME [8,28,29], AALLME [9], gas chromatography coupled to mass spectrometry (GC-MS) [10,28] or tandem mass spectrometry (GC-MS/MS) [11], liquid chromatography coupled to tandem mass spectrometry (LC-MS/MS) [8] or quadrupole time-of-flight mass spectrometry (LC-qTOF/MS) [27], HPLC-ultraviolet detection (HPLC-UV) [9], as well as HPLC-fluorescence-ultraviolet detection (HPLC-FLD-UV) [29]. 

We would like to emphasize that the absolute recovery data obtained in the present study are similar or better than the compared microextraction-based approaches. Additionally, the precision values achieved present the same magnitude, apart from the SPME/GC-MS/MS or DLLME/LC-MS/MS methodologies (RSD ≤ 44%). Finally, and as expected, the proposed analytical approach presents lower sensitivity when compared to MS or tandem MS, but much better sensitivity when compared to similar instrumental systems.

In short, our attained figures of merit compare very favourably with other microextraction-based methodologies already reported in the literature, which makes the present analytical approach a promising alternative for future applications. Furthermore, the use of mixed carbon materials allows for fast and easy tailoring of the sorbent coating according the target compounds. Finally, the proposed miniaturized analytical approach employs negligible amounts of organic solvents, which is in accordance with green analytical principles. This information was added to the manuscript.

### 2.5. Application to Environmental Water Matrices

**Figure 3:**
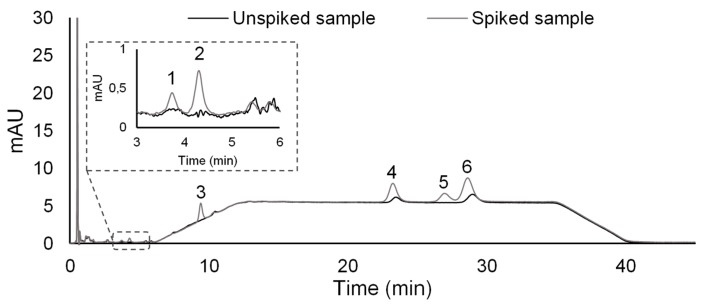
Chromatogram obtained from an assay performed on spiked (25.0 µg L^−1^) and unspiked wastewater samples obtained by BAµE(R, 12.5%/CN1, 87.5%)-µLD/HPLC-DAD under optimized experimental conditions. 1. BSA; 2. OHBT; 3. BT; 4. MeBT; 5. BTh; 6. OHBTh.

The proposed analytical approach was also applied to four real water matrices, including rain, waste, tap, and estuarine water samples, through the standard addition method (SAM). After sample filtration, four working standards (25.0 to 100.0 µg L^−1^) were used for spiking the analysed matrices and blank assays (without spiking). This is a similar range found in the literature for the determination of these compounds in aqueous media using HPLC-DAD systems [9,29]. The data obtained demonstrate (Table 3) that good linearities were also obtained for all four real water matrices (*r*^2^ ≥ 0.9907). However, it must be emphasized that significant matrix effects were noted. In order to compare this effect from the different assayed samples, we included Table S2 where we show the obtained regression equations from ultrapure water assays. As it can be seen from Table S3, in general, the most extensive matrix effects were obtained for the wastewater, which was expected, as this type of sample usually has large amount of contaminants. The remaining samples, on average, presented the same order of matrix effects. It must also be emphasized that, in general, BSA and OHBT presented the highest matrix effects. This can be attributed to the more polar nature of these compounds, making them more hydrophilic in comparison with the other target compounds. Nevertheless, by applying the developed methodology through SAM, it was possible to determine the presence of MeBT (11.9 µg L^−1^) and OHBTh (10.8 µg L^−1^) in the wastewater sample (Figure 3), similar to values already reported in literature for similar matrices [1,8,29].

## 3. Experimental

### 3.1. Standards and Materials

All reagents and solvents were of analytical grade and used with no further purification. MeBT (≥ 98.0%), BT (≥ 99.0%), BSA (≥ 98.0%) and BTh (≥ 97.0%) were from Alfa Aesar (Karlsruhe, Germany). OHBTh (≥ 95.0%) and OHBT (≥ 98.0%) were purchased from Thermo Fisher Scientific (Winsford, U.K.). The solvents used were HPLC-grade methanol (MeOH, 99.8%) and acetonitrile (ACN, 99.8%) obtained from Thermo Fisher Scientific (Winsford, U.K.). Sodium chloride was purchased from Merck (NaCl, 99.5%; Darmstadt, Germany). Sodium hydroxide pellets was obtained from AnalaR (NaOH, 98.0%; BDH chemicals, Poole, U.K.) and hydrochloric acid (HCl, 37%) was provided from Panreac (Barcelona, Spain). Ultra-pure water was obtained from the Milli-Q water purification systems (Merck Millipore, Darmstadt, Germany). The commercial ACs, i.e., SX1 (surface area 900 m^2^/g and pH_PZC_ 8.4), CN1 (surface area 1,400 m^2^/g and pH_PZC_ 6.4), CA1 (surface area 1,400 m^2^/g and pH_PZC_ 2.2), B Test EUR (surface area 1500 m^2^/g and pH_PZC_ 6–8) and SX PLUS (surface area 1100 m^2^/g and pH_PZC_ 6–8) were from Cabot Norit and supplied by Salmon & Cia (Lisbon, Portugal); and R (surface area 937 m^2^/g and pH_PZC_ 6.5), purchased from Riedel-de Haën (Seelze, Germany).

Stock solutions of individual analytes (1000.0 mg L^−1^) used for the working standard mixture were prepared in MeOH and stored at −20 °C and renewed every month. Working standard mixtures of 5.0 mg L^−1^ were daily prepared in MeOH and used for spiking sample assays. For instrumental calibration, standard mixtures were prepared in MeOH by appropriate dilution from previous stock solutions.

### 3.2. BAµE-µLD Assays

The BAµE devices (7.5 mm in length and 3 mm in diameter) were lab-made and prepared according to previous works [19,20,30]. The BAµE devices were cleaned with MeOH and ultra-pure water before use. Typical assays were performed in sampling flasks having 25 mL of ultra-pure water spiked with 100 µL of the working standard mixture to obtain a final concentration of 20.0 µg L^−1^, followed by the introduction of the BAµE device previously coated with the powdered sorbent and a conventional Teflon stir bar. The assays were performed in a multipoint agitation plate (Variomag, Langenselbold, Germany) at room temperature. To evaluate the BAµE efficiency, systematic studies were performed in triplicate for the optimization of several parameters, such as sorbent phase type (ACs and AC mixtures), equilibrium time (1, 3, and 16 h), stirring rate (750, 1000, and 1250 rpm), pH (2.1, 5.5, 8.0, 9.5, and 11.0), organic modifier (MeOH; 0%, 5%, 10%, and 15%, *v*/*v*), and ionic strength (NaCl; 0%, 5%, 10%, 15%, and 20%, *w*/*v*). After the microextraction stage, the BAµE devices were removed from the samples with clean tweezes and placed into a vial containing a glass insert filled with 100 µL of the striping solvent inside a 2 mL vial, ensuring their total immersion prior to ultrasonic treatment (42 ± 2.5 kHz, 100 W, Branson 3510, Carouge, Switzerland) at room temperature. For the back-extraction stage, ACN, MeOH, and mix (MeOH/ACN, 1/1, *v*/*v*) were the desorption solvents tested, using several periods of time (30, 45, and 60 min) under ultrasonic treatment. Subsequently, the BAµE devices were removed and the vials were sealed and placed into the auto-sampler tray for HPLC-DAD analysis. For the AC-based mixture assays, accurately weighted amounts of R/CA1 (25%/75% and 50%/50% *w*/*w*) and R/CN1 (12.5%/87.5%, 25%/75%, 37.5%/62.5%, and 75%/25% *w*/*w*) were homogenized in a shaker for 15 min prior to the same preparation described previously.

For method validation experimental purposes, 25 mL of ultra-pure water were spiked with appropriate volumes of working standard mixtures to reach the desired concentrations, and then the assays were performed in triplicate under optimized experimental conditions. Blank assays were also performed in triplicate using the procedure above without spiking. The sensitivity of the methodology was checked through the limits of detection (LODs) and quantification (LOQs), calculated with a signal-to-noise ratio (S/N) of 3/1 and 10/1, respectively. The carryover was also checked by a series of replicates. The present methodology was also evaluated through intraday and interday repeatability assays and calculated as relative standard deviation (RSD) on five and nine assays, respectively.

### 3.3. Assays on Real Water Matrices

The water samples were collected in the metropolitan area of Lisbon (Lisbon, Portugal). The rain and tap water were collected in the Campo Grande garden. The estuarine water was collected from the Tagus estuary near Cais do Sodré docking station. The wastewater sample was obtained from the Alcântara wastewater treatment plant after primary decantation and filtration. All samples were collected in clean amber glass bottles and filtered with paper filters (125 mm of diameter, 10–13 µm of pore size, Cat No 1001 125, Whatman; Maidstone, U.K.) and kept refrigerated at −20 °C until being used. Whenever possible, the samples were analysed on the same day. The standard addition method (SAM) was applied for real matrices assays using the optimized procedure, at 4 spiking concentrations (25.0–100.0 µg L^−1^), including the assays without spiking, in triplicate.

### 3.4. Instrumental Set-Up

HPLC-DAD analysis were carried out on a benchtop Agilent 1100 series LC chromatographic system (Agilent Technologies, Waldbronn, Germany) equipped with a vacuum degasser (G1322A), autosampler (G1313A), thermostated column compartment (G1316A), quaternary pump (G1311A), and a diode array detector (G1315B). The data acquisition and system control were performed by the software LC3D ChemStation (version Rev.A.10.02[1757], Agilent Technologies, Waldbronn, Germany). Analyses were performed on a Mediterranea Sea C18 column, 150.0 × 4.6 mm, 2.6 µm particle size (Teknokroma, Barcelona, Spain). The samples were analysed using a gradient mobile phase consisting of ultra-pure water (solvent A) and MeOH (solvent B). The employed elution gradient was 0 min: 5% B; 4 min: 5% B; 10 min: 10% B; 33 min: 10% B; 38 min: 5% B; 43 min: 5% B. All solvents were previously filtered (125 mm in diameter, 10–13 µm in pore size, Cat. No. 1001 125, Whatman, Maidstone, U.K.) to remove suspended particles, if any. The detector was set at 210 nm and the column temperature at 25 °C. The injection volume was 5 µL with a draw speed of 200 µL min^−1^ and the flow rate was set at 0.75 mL min^−1^. For identification purposes, standard addition was used by spiking the samples with pure standards, as well as by comparing the relative retention time and peak purity with the UV/vis spectral reference data. For quantification purposes, calibration curves using the external standard methodology were performed. The recovery data of all performed assays were calculated through the comparison of the average peak areas of the extracted analytes with standard controls. Peak areas were obtained by integration of each target compounds’ corresponding peak using the mentioned software. The sensitivity of the instrumental system was checked through the LODs and LOQs calculated with S/N of 3/1 and 10/1, respectively. The instrumental precision was evaluated by consecutively injecting a standard mixture (*n* = 6, 1.0 mg L^−1^).

## 4. Conclusions

The proposed methodology, using carbon-based phase mixtures as coating phases for bar adsorptive microextraction, followed by microliquid desorption and high performance liquid chromatography-diode array detection (BAµE-µLD/HPLC-DAD), enhanced the performance of determination of traces of BSA, OHBT, BT, MeBT, BTh, and OHBTh chemicals in rain, waste, tap, and estuarine water matrices. Under a particular carbon-based phase mixture (R, 12.5%/CN1, 87.5%), the most important experimental parameters affecting the microextraction and back-extraction stages were fully optimized and validated, resulting in remarkable analytical data. Apart from several advantages of BAµE technology, the proposed approach allows for tailoring for the best mixture phase selectivity with convenient porosity, texture, and surface chemistry characteristics, which can be a major advantage when simultaneously analysing compounds with distinct physicochemical properties. To the best of our knowledge, this is the first work that employs carbon-based mixed phases for a microextraction-based methodology to monitor traces of BTRs, BTs, and BSDs in environmental water matrices.

## Figures and Tables

**Table 1:** Limits of detection (LODs) and limits of quantification (LOQs) as well as intraday and interday repeatability of recovery assays obtained for BSA, OHBT, BT, MeBT, BTh, and OHBTh chemicals by BAµE(R, 12.5%/CN1, 87.5%)-µLD/HPLC-DAD under optimized experimental conditions.

Compound	LODs(μg L^−1^)	LOQs(μg L^−1^)	Recovery (%) ± RSD (%)	
			Intraday Assays (*n* = 5)	Interday Assays (*n* = 9)
BSA	1.0	3.3	59 ± 3	58 ± 6
OHBT	1.4	4.0	38 ± 9	38 ± 9
BT	1.0	3.3	56 ± 3	56 ± 9
MeBT	1.2	4.0	59 ± 2	59 ± 2
BTh	1.2	4.0	57 ± 6	56 ± 9
OHBTh	1.0	3.3	53 ± 4	53 ± 3

**Table 2:** Comparison of the proposed method with other microextraction-based approaches previously reported in the literature for the determination of benzotriazoles (BTRs), benzothiazoles (BTs), and benzenesulfonamide derivatives (BSDs) in environmental water matrices.

Microextraction Technique	SPME	DLLME	SBSE	DLLME	AALLME	DLLME	SPME	BAμE
**Instrumental System**	GC-MS/MS	LC-MS/MS	GC-MS	HPLC-FLD-UV	HPLC-UV	GC-MS	LC-qTOF/MS	HPLC-DAD
**Sample Type**	Tap, river, and effluent waste water	Ground, river, influent, and effluent waste water	Influent waste water	Tap, river, industrial waters, as well as effluent and influent wastewaters	Tap, lake, river, as well as effluent and influent wastewaters	Tap and effluent wastewaters	Tap, as well as influent and effluent wastewaters	Tap, estuarine, rain, and effluent wastewaters
**Sorbent or Solvent Phase Used for Microextraction**	Polyacrylate	Chloroform/Carbon tetrachloride/ACN	Polyacrylate	Tri-*n*-butylphosphate/methanol	1-Hexanol	Toluene/ACN	Polyethersulfone	Mixed ACs
**LODs (μg L^−1^)**	0.0001–7.5	0.04–0.75	0.256	0.04–2.2	0.8–1.4	0.007–0.080 ^a^	0.005–0.1 ^a^	1.0–1.4
**Linear Range(μg L^−1^)**	1.0–100.0	0.01–50.0	0–10.0	2.4–536.1	5.0–10000.0	0.05–20.0	0.1–50.0	5.0–120.0
**Precision (%)**	≤ 24.6	≤ 44.0	≤ 9.8	≤ 8.4	≤ 7.8	≤ 8.0	≤ 8.0	≤ 9.3
**Absolute Recovery (%)**	n.a.	5.0–42.0	n.a.	67.4–97.1	n.a.	24.0–46.0	n.a.	37.9–59.2
**Reference**	[11]	[8]	[10]	[29]	[9]	[28]	[27]	This work

^a^ These values correspond to the method LOQs. The LODs are not available.

**Table 3:** Calibration parameters, including *r*^2^, for the four real water samples spiked between 25.0 and 100.0 μg L^−1^ and obtained for BSA, OHBT, BT, MeBT, BTh, and OHBTh chemicals by BAµE(R, 12.5%/CN1, 87.5%)-µLD/HPLC-DAD through the standard addition method (SAM) approach under optimized experimental conditions.

Compounds	Slope	Intercept	*r* ^2^		Slope	Intercept	*r* ^2^
		Tap water		Estuarine water			
BSA	0.3000	0.0788	0.9938		0.2600	0.0676	0.9913
OHBT	0.3000	0.0672	0.9920		0.2600	0.0608	0.9907
BT	1.5600	1.1600	0.9989		5.1200	1.3560	0.9927
MeBT	1.8400	1.6944	0.9987		0.4200	3.1112	0.9992
BTh	0.5000	2.0740	0.9937		0.2000	2.1520	0.9962
OHBTh	1.9800	5.5472	0.9957		0.8800	5.1488	0.9993
		Rainwater				Waste water	
BSA	0.2800	0.0692	0.9926		0.2400	0.0680	0.9934
OHBT	0.4400	0.0992	0.9907		0.0800	0.0314	0.9923
BT	2.2000	1.4620	0.9973		4.2000	1.1480	0.9932
MeBT	0.6400	3.0044	0.9988		31.0000	2.6044	0.9991
BTh	0.2000	2.0480	0.9970		0.8000	2.0320	0.9995
OHBTh	0.5000	4.6128	0.9989		47.1200	4.3584	0.9984

## Supplementary Materials

The following are available online at https://www.mdpi.com/1420-3049/25/9/2133/s1, Table S1: chemical properties of the target compounds, Table S2: instrumental results, Table S3: calibration parameters using ultra-pure water; Figure S1: effect of solvent type and sonication time on the microextraction performance; Figure S2: effect of stirring speed on the microextraction performance; Figure S3: effect of MeOH and NaCl addition on the microextraction performance.
