# Supplementary material for: Bar Adsorptive Microextraction Coated with Carbon-Based Phase Mixtures for Performance-Enhancement to Monitor Selected Benzotriazoles, Benzothiazoles, and Benzenesulfonamides in Environmental Water Matrices
Source: Molecules. 2020 May 2;25(9):2133. doi: 10.3390/molecules25092133 (PMC7248745; doi:10.3390/molecules25092133)
Supplement: Supplementary file 1 [file molecules-25-02133-s001.pdf]

Supplementary material

Table 1. Chemical structures, pKa values as well as the log values in relation to the sample pH of each compound under study, obtained by dedicated software (Marvin 6.2.2, 2014, ChemAxon, http://www.chemaxon.com).

Compound	Chemical structure	pKa value	Sample pH	Log D value
**Benzenesulfonamide (BSA)**	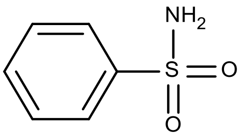	10.24	2.00	0.58
			5.50	0.58
			8.00	0.58
			11.00	-0.03
**1H-1,2,3-benzotriazol-1-ol (OHBT)**	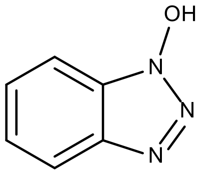	6.88	2.00	0.63
			5.50	0.61
			8.00	-0.50
			11.00	-1.74
**1H-1,2,3-benzotriazole (BT)**	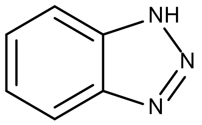	8.63	2.00	1.29
			5.50	1.30
			8.00	1.21
			11.00	-0.24
**5-methyl-1H-1,2,3-benzotriazole (MeBT)**	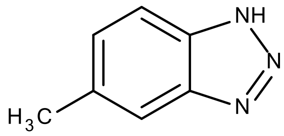	8.85	2.00	1.77
			5.50	1.81
			8.00	1.76
			11.00	0.31
**1,3-benzothiazole (BTh)**	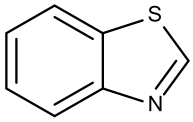	2.25	2.00	1.88
			5.50	2.11
			8.00	2.11
			11.00	2.11
**1,3-benzothiazol-2-ol (OHBTh)**	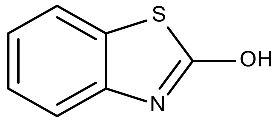	11.27	2.00	2.49
			5.50	2.49
			8.00	2.36
			11.00	1.08

Table 2. Retention times (RT), resolution values, LODs, LOQs and r^2 obtained for the target compounds under optimized instrumental conditions.

Compound	RT (min)	Resolution [a]	LODs (mg L^{-1})	LOQs (mg L^{-1})	$r^{2\,b}$
**BSA**	4.6	n.a.	0.10	0.330	0.9994
**OHBT**	5.1	2.0	0.01	0.033	0.9996
**BT**	10.8	17.0	0.05	0.165	0.9994
**MeBT**	24.8	20.7	0.05	0.165	0.9992
**BTh**	28.9	3.7	0.05	0.165	0.9986
**OHBTh**	31.1	1.8	0.05	0.165	0.9990

[a]Calculated in accordance with the RT of the previous peak.

[b]Linear range between 0.25 and 5.0 mg L^{-1} (6 levels).

Table 3. Calibration parameters, including r^2, slopes and intercept obtained for BSA, OHBT, BT, MeBT, BTh and OHBTh chemicals by BAμE(R, 12.5 %/CN1, 87.5 %)-μLD/HPLC-DAD in ultrapure water samples, under optimized experimental conditions.

Compounds	Linear Range (μg L^{-1})	Slope	Intercept	r^2
BSA	5.0 – 120.0	3.1384	0.0906	0.9974
OHBT		9.3813	0.5192	0.9974
BT		7.8438	1.4693	0.9967
MeBT		1.8446	3.3428	0.9971
BTh		1.4750	2.3074	0.9980
OHBTh		11.6360	5.1999	0.9964

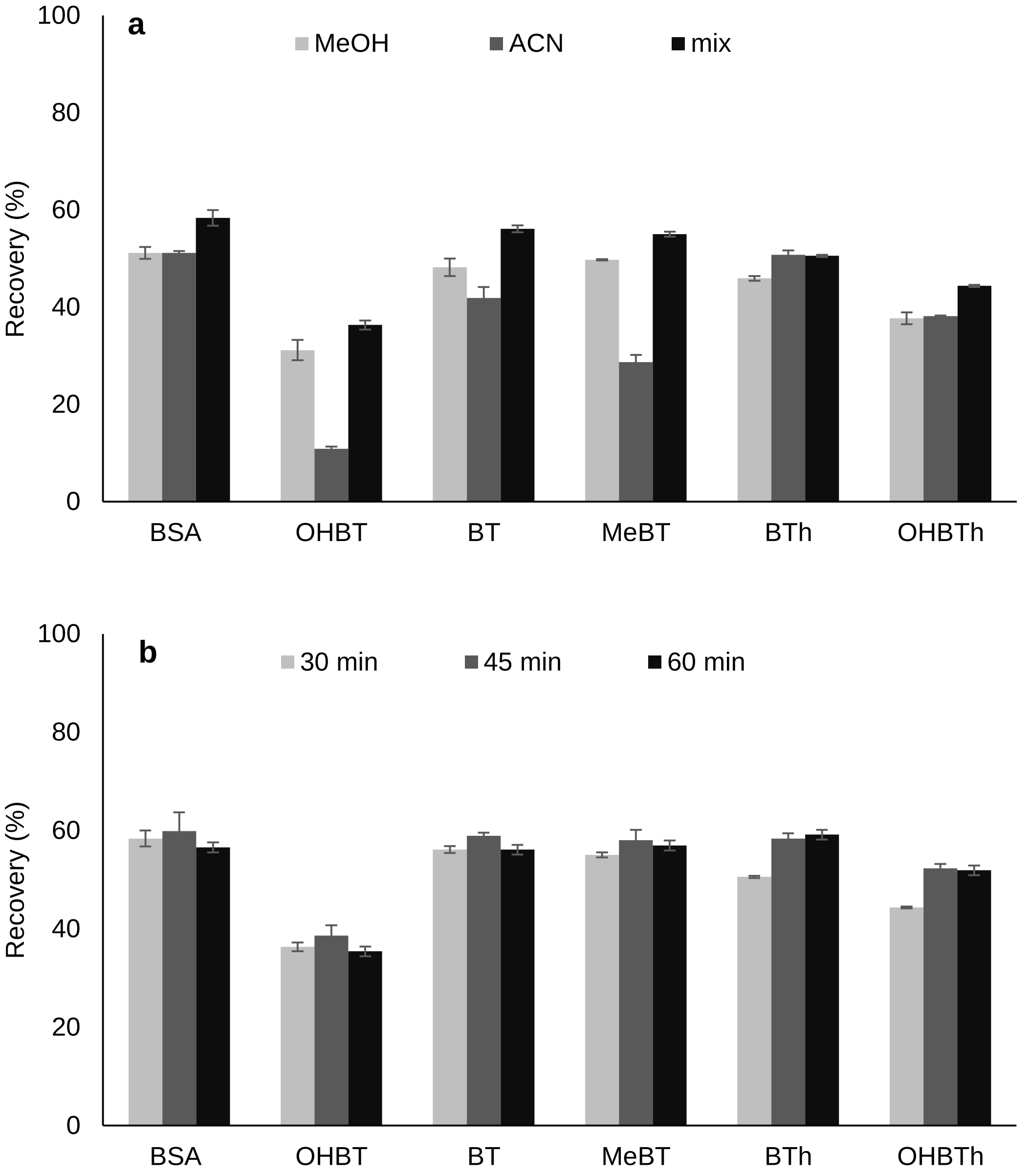

Figure 1. Effect of solvent type (a) and sonication time (b) on the microextraction of the six micropollutants from aqueous media obtained by BAμE-μLD/HPLC-DAD. The error bars represent the standard deviation of three replicates.

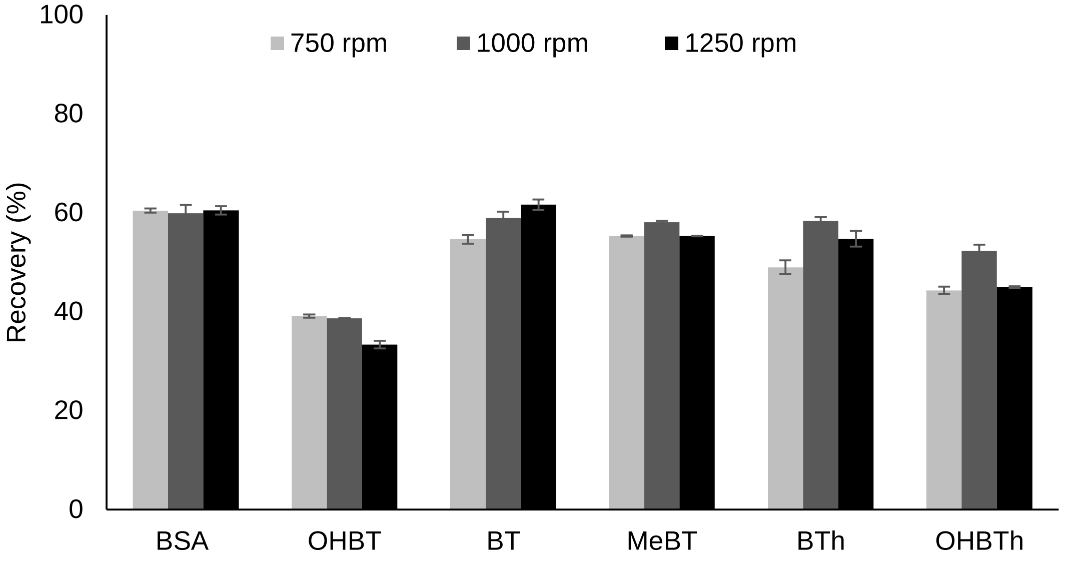

Figure 2. Effect of stirring rate on the microextraction of the six micropollutants from aqueous media obtained by BAμE-μLD/HPLC-DAD. The error bars represent the standard deviation of three replicates.

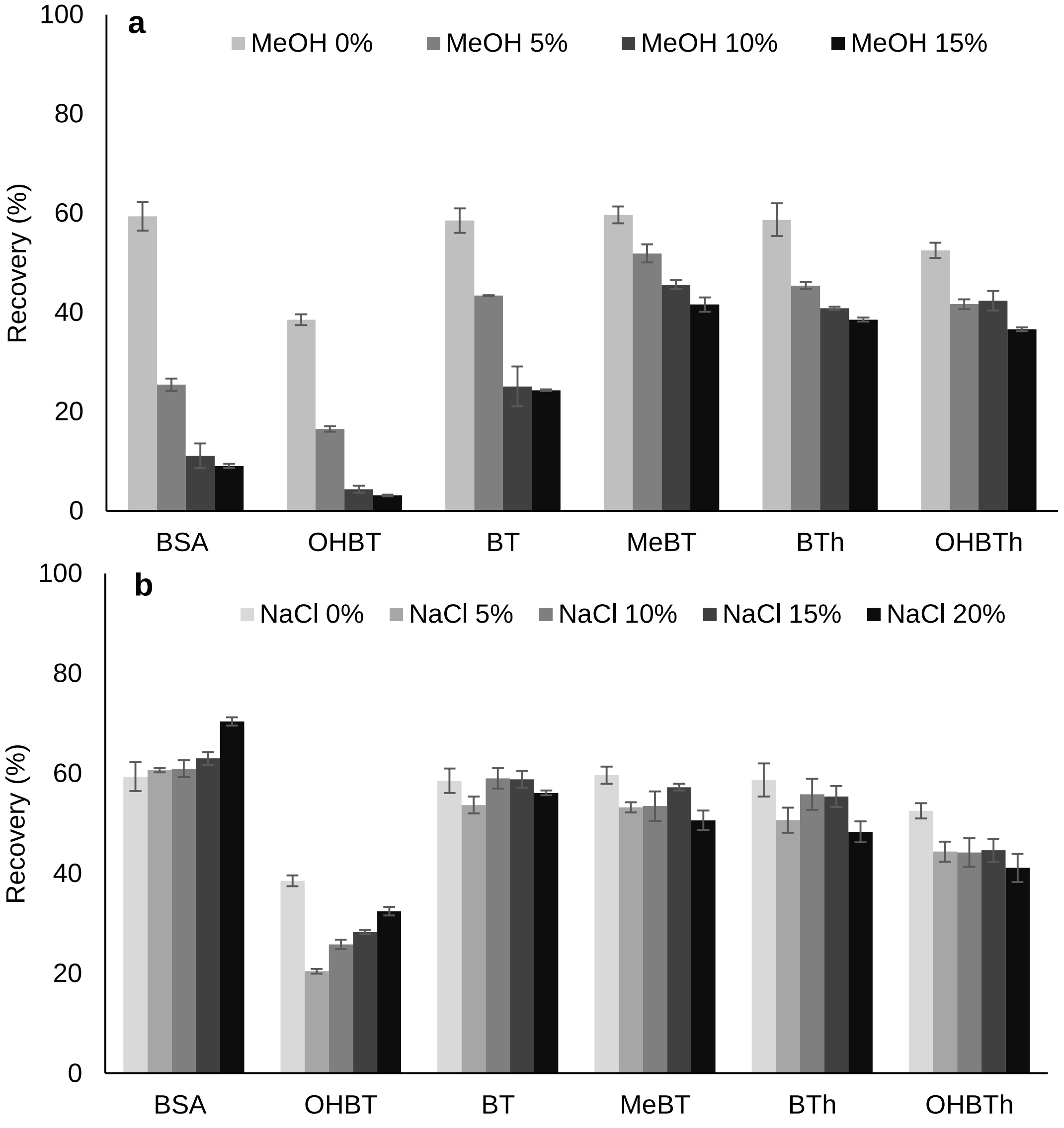

Figure 3. Effect of MeOH (a) and NaCl (b) addition on the microextraction of the six micropollutants from aqueous media obtained by BAμE-μLD/HPLC-DAD. The error bars represent the standard deviation of three replicates.
